# Supplementary material for: Fundamental and Advanced Therapies, Vaccine Development against SARS-CoV-2
Source: Pathogens. 2021 May 21;10(6):636. doi: 10.3390/pathogens10060636 (PMC8224379; doi:10.3390/pathogens10060636)
Supplement: Supplementary file 1 [file pathogens-10-00636-s001.zip › pathogens-1205099-supplementary.pdf]

Table S1. Comparison of different vaccine types (WHO, 2020; Zimmer et al., 2020)

Vaccine name	Developer	Immunogen	Doses	Age	Contraindications	Efficacy	Protection starts	Storage mounts (M)
**Comirnaty**	BioNTech Pfizer	mRNA	2 (21 days apart)	16+	Allergy to ingredients	95 %	7 days	6 M (-90°C to -60°C)
**COVID-19 Vaccine Moderna**	NIAID Moderna	mRNA	2 (28 days apart)	18+	Allergy to ingredients	94.1 %	14 days	7 M (-25°C to -15°C)
**Vaxzevria**	SII SKBio	adenovirus (DNA)	2 (4-12 weeks apart)	18+	Hypersensitivity to ingredients	82.4 %	15 days	6 M (2°C to 8°C)
**COVID-19 Vaccine Janssen**	Johnson & Johnson	adenovirus (DNA)	1	18+	Hypersensitivity to ingredients	72 %	28 days	3 M (2°C to 8°C)
**Sputnik V**	GRIEM	adenovirus (rAd26 and rAd5)	2 (21 days apart)	18+	Severe allergic reaction	91.6 %	No data available	6 M (-25°C to -15°C)
